# Inhibition of Cathepsins B Induces Neuroprotection Against Secondary Degeneration in Ipsilateral Substantia Nigra After Focal Cortical Infarction in Adult Male Rats

**DOI:** 10.3389/fnagi.2018.00125

**Published:** 2018-05-09

**Authors:** Xialin Zuo, Qinghua Hou, Jizi Jin, Xiaohui Chen, Lixuan Zhan, Yanyan Tang, Zhe Shi, Weiwen Sun, En Xu

**Affiliations:** ^1^Institute of Neurosciences and Department of Neurology, The Second Affiliated Hospital, Guangzhou Medical University, The Key Laboratory of Neurogenetics and Channelopathies of Guangdong Province and the Ministry of Education of China, Guangzhou, China; ^2^Department of Neurology, The Seventh Affiliated Hospital, Sun Yat-sen University, Shenzhen, China; ^3^Department of Emergency, The Second Affiliated Hospital, Guangzhou Medical University, Guangzhou, China

**Keywords:** cerebral infarction, striatum, substantia nigra, cathepsin B, CA-074Me, secondary degeneration

## Abstract

Stroke is the leading cause of adult disability in the world. In general, recovery from stroke is incomplete. Accumulating evidences have shown that focal cerebral infarction leads to dynamic trans-neuronal degeneration in non-ischemic remote brain regions, with the disruption of connections to synapsed neurons sustaining ischemic insults. Previously, we had reported that the ipsilateral striatum, thalamus degenerated in succession after permanent distal branch of middle cerebral artery occlusion (dMCAO) in Sprague-Dawley (SD) rats and cathepsin (Cath) B was activated before these relay degeneration. Here, we investigate the role of CathB in the secondary degeneration of ipsilateral substantia nigra (SN) after focal cortical infarction. We further examined whether the inhibition of CathB with L-3-trans-(Propyl-carbamoyloxirane-2-carbonyl)-L-isoleucyl-L-proline methyl ester (CA-074Me) would attenuate secondary degeneration through enhancing the cortico-striatum-nigral connections and contribute to the neuroprotective effects. Our results demonstrated that secondary degeneration in the ipsilateral SN occurred and CathB was upregulated in the ipsilateral SN after focal cortical infarction. The inhibition of CathB with CA-074Me reduced the neuronal loss and gliosis in the ipsilateral SN. Using biotinylated dextran amine (BDA) or pseudorabies virus (PRV) 152 as anterograde or retrograde tracer to trace striatum-nigral and cortico-nigral projections pathway, CA-074Me can effectively enhance the cortico-striatum-nigral connections and exert neuroprotection against secondary degeneration in the ipsilateral SN after cortical ischemia. Our study suggests that the lysosomal protease CathB mediates the secondary damage in the ipsilateral SN after dMCAO, thus it can be a promising neuroprotective target for the rehabilitation of stroke patients.

## Introduction

Stroke is the leading cause of adult disability in the world. In general, recovery from stroke is incomplete. Therefore, the improvement of long-term poor outcomes for stroke patients is significant. Accumulating evidences have shown that focal cerebral infarction leads to neuropathologic damages not only at lesion site, but also in nonishemic remote brain regions that have synaptic connections with the primary lesion (Uchida et al., 2010; Zhang et al., 2012a; Ohe et al., 2013; Fernández-Andújar et al., 2014; Jones et al., 2015; van Etten et al., 2015). This phenomenon, termed secondary degeneration, has been reported to be associated with sustained dementia, the development of vascular Parkinson’s syndrome and worsen stroke outcomes (Kirton et al., 2007; Domi et al., 2009; DeVetten et al., 2010; Rodriguez-Grande et al., 2013; Baron et al., 2014). Ohe et al. (2013) investigated the background and features of patients exhibiting secondary degeneration of substantia nigra (SN) in 43 patients with MRI. These patients included 7 patients (16%) with striatal infarction and 36 patients (84%) with striatal and cortical infarctions. Hyperintense regions in the mesencephalic SN were observed in all patients after 7–28 days on diffusion weighted imaging (DWI) or fluid-attenuated inversion recovery (FLAIR) and T2-weighted MRI (T2), indicating that secondary degeneration of the mesencephalic SN also occur in human cerebral infarction (Ohe et al., 2013).

Previously, we have demonstrated that secondary damage occurred in the ipsilateral ventroposterior nucleus (VPN) of the thalamus after focal cortical infarction (Zuo X. et al., 2016). This phenomenon was also shown by many other groups (Wang et al., 2007; Zhang et al., 2011; Xing et al., 2012, 2014). SN is another brain structure located in the midbrain that is usually vulnerable to secondary degeneration after middle cerebral artery occlusion (MCAO) in animal experiments. Owing to its delayed occurrence, secondary damage can be a promising target for new neuroprotective strategies beyond the narrow time window for acute stroke therapy (Prinz et al., 2015). Thus, this may be of significant clinical value.

To date, the mechanisms of occurrence of secondary degeneration in remote areas after ischemic stroke remain unclear. Numerous plausible explanations have been proposed for the secondary neuronal damage, including retrograde degeneration (Wang et al., 2007), inflammation (Block et al., 2005), neurotoxicity and neuron inhibitory factors, such as β-Amyloid (Aβ) and Nogo-A (Wang et al., 2007; Zhang et al., 2011), oxidative damage (He et al., 2007), autophagy (Xing et al., 2012, 2014; Zhang et al., 2012a), and others (Zhang et al., 2012b).

Lysosomes are membrane-enclosed organelles, which contain hydrolytic enzymes that are capable of breaking down proteins, nucleic acids, and lipids. They play critical roles in signal transduction, cell metabolism, exocytosis, plasma membrane repair, and cell death (Boya and Kroemer, 2008; Settembre et al., 2013). Lysosomal membranes are vulnerable to insults such as ischemia and reperfusion (I/R) injury and oxidative stress (Aits and Jäättelä, 2013; Appelqvist et al., 2013; Lipton, 2013; Repnik et al., 2014). Loss of membrane integrity will result in the release of cysteine cathepsins (Cath) into the cytoplasm. As well as most of lysosomal proteases, Cath, in principle poses little danger, are inactive at neutral pH. However, some Cath, such as CathB, D, and L, remain active at neutral pH, thus triggering potential cell death (Aits and Jäättelä, 2013; Serrano-Puebla and Boya, 2016).

CathB is the most abundant neuronal lysosomal protease in the brain. It distributes almost exclusively within neurons and scatters throughout the cytosol, dendrites, and synapses (Hou et al., 2010; Graber et al., 2004). Under pathological stimulations, CathB is reported to escapes from lysosome into the cytoplasm or extracellular matrix, causing cellular autolysis, apoptosis, excessive autophagy, and even damage to neighboring cells (Graber et al., 2004; Tsubokawa et al., 2006; Kilinc et al., 2010; Wang et al., 2011; Xu et al., 2014; Hook et al., 2015). However, it remains to be elucidated whether CathB is involved in the secondary degeneration from cortex to SN after focal cortical infarction.

To address these knowledge gaps, we conducted this study to investigate the role of CathB in the secondary degeneration of ipsilateral SN after permanent distal branch of middle cerebral artery occlusion (dMCAO) in Sprague-Dawley (SD) rats. We further examined whether the inhibition of CathB with L-3-trans-(Propyl-carbamoyloxirane-2-carbonyl)-L-isoleucyl-L-proline methyl ester (CA-074Me) would attenuate secondary degeneration through enhancing the cortico-striatum-nigral connections and contribute to the neuroprotective effects against cortical ischemia in adult rats.

## Materials and Methods

**FIGURE 1:**
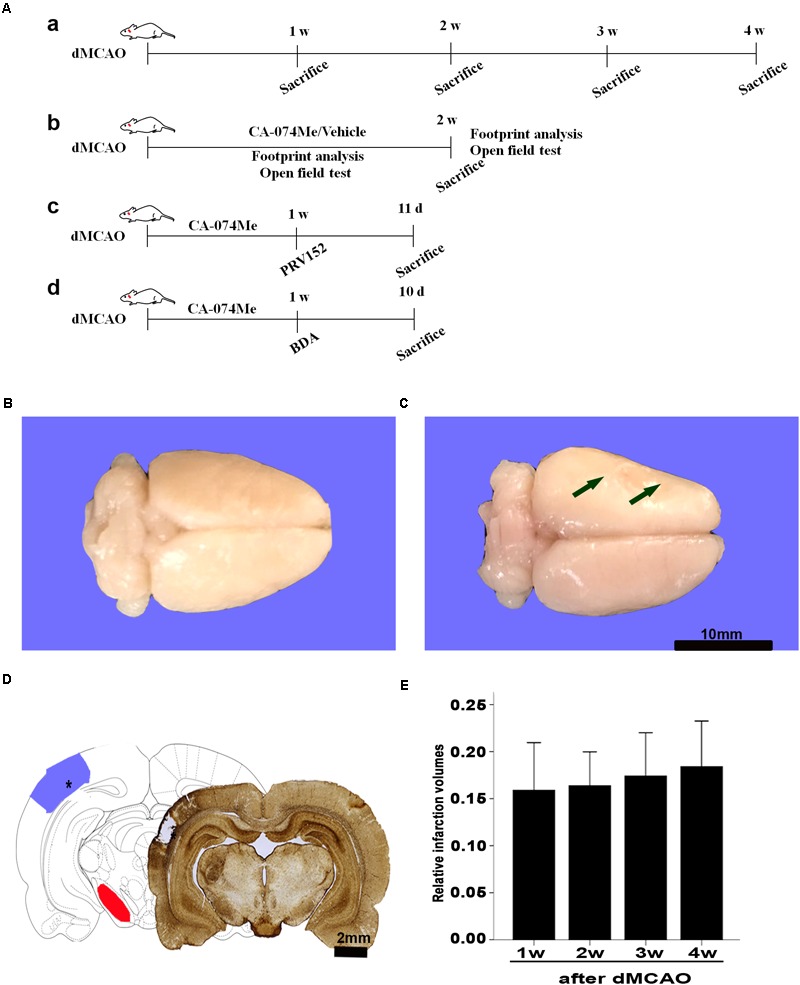
Focal cortical infarction is induced by dMCAO in rats. **(A)** Study design/timeline of experiments. **(a)** Rats were sacrificed for immunohistochemistry and Western blotting at 1–4 weeks after dMCAO or sham operation; **(b)** Footprint analysis and open field test were performed at 1 and 2 weeks, rats were sacrificed at 2 weeks in the vehicle group and CA-074Me group after dMCAO or sham operation; **(c)** PRV-152 was injected into the ipsilateral SNr at 1 week. Rats were sacrificed at 11 days in sham-operated group, dMCAO group, and CA-074Me group. **(d)** BDA was injected into the ipsilateral SNr at 1 week, and rats were sacrificed at 10 days in sham-operated group, dMCAO group, and CA-074Me group. **(B,C)** Gross brain morphology was indicated in the sham-operated and dMCAO groups at 2 weeks after dMCAO. **(D)** GFAP/DAB-staining confirmed the existence of focal cerebral infarction in the dMCAO group. **(E)** Relative infarction volumes were evaluated at 1, 2, 3, and 4 weeks after dMCAO (*n* = 7, 6, 7, and 6, respectively). The arrow in panel B and the star in panel C indicated cortical infarction after dMCAO. Scale bar in panel B and C is 10 and 2 mm, respectively; w, week; d, day.

Experiments were performed on adult male SD rats weighing 280–320 g (Southern Medical University, Guangzhou, China). Rats for the experiment were housed under standard temperature (22°C ± 1°C) a 12-h light/dark controlled environment with free access to food and water. Weight gain and health condition of rats are comparable among different groups. All animal procedures were performed in accordance with Animal Research: Reporting *In Vivo* Experiments (ARRIVE) guidelines and were approved and monitored by the Animal Care and Use Committee of Guangzhou Medical University. All efforts had been made to minimize the number of animals used and the suffering of the animals. An overall study design/timeline has been shown in **Figure 1A**.

### Animal Model

Permanent occlusion of distal branch of middle cerebral artery (dMCAO) was performed using an electrocoagulation methodology described previously (Wang et al., 2012; Zuo X. et al., 2016). Briefly, rats were placed in the anesthesia induction box supplied with 3–4% isoflurane in 100% oxygen. Anesthesia was maintained with 1.5–2.5% isoflurane in 100% oxygen, delivered through a nose mask (SurgiVet, Waukesha, WI, United States) during the surgical procedure. The distal striatal branch of MCA was exposed and occluded by unipolar electrocoagulation under an operating microscope. Sham-operated animals were performed with the same surgical procedures except for electrocoagulation of the distal MCA. Rectal temperature of the animals was monitored and maintained at approximately 37°C throughout the procedure. After surgery, the rats were allowed to wake up and evaluated the neurological status as described by previously (Rodriguez-Grande et al., 2013). Rats neither with neurologic deficit nor with cortical infarction were excluded from this study.

### Histology

Animals were intracardially perfused with normal saline, followed by 4% paraformaldehyde (PFA) in phosphate-buffered saline (PBS) (0.01M, pH 7.4) under anesthesia with 10% chloral hydrate administered intraperitoneally. The brains were postfixed for 12 h in 4% PFA. Then postfixed brains were immersed in 10, 20, and 30% sucrose in the same fixative for cytoprotection. Coronal tissue blocks (bregma 1.7 to -5.8 mm) were cut on a freezing microtome (Leica CM1950, Heidelberg, Germany) into 30-μm-thick sections. Fluoro-Jade B (FJB) staining was used to selectively label the degenerating neurons. In brief, sections were mounted on gelatin-coated glass slides, air-dried, and were then immersed in 100% ethanol for 3 min followed by 70% ethanol and water for 1 min each. Sections were then transferred to 0.06% potassium permanganate for 15 min. After 2 more rinses, they were placed in 0.001% FJB (Millipore, Bedford, MA, United States) in 0.1% acetic acid for 60 min at room temperature in the dark. After that, they were water-washed and mounted with distrene plasticizer xylene (Sigma). The FJB-stained sections were examined under a fluorescence microscope (Leica Microsystems, Wetzlar, Hessen, Germany). Some of slides of FJB staining were counterstained with CathB immunofluorescent staining (Zuo X.L. et al., 2016). After mounting, sections were immediately observed under a fluorescence microscope.

For infarct volume evaluation, glial fibrillary acidic protein (GFAP)/diaminobenzidine (DAB)-staining experiment was carried out, and a series of cover sections on both sides of all brain tissue blocks of each animal were picked out for this purpose. Each stained section was photographed. The territory of infarction and the total area of brain were outlined and quantitated digitally using ImageJ software (NIH, Bethesda, MD, United States). Then, the infarction volume was calculated by multiplying the block thickness, and the percentage volume of infarction was normalized by the volume of the contralateral non-ischemic hemisphere.

### Immunohistochemistry

The rats were divided into four groups randomly for immunohistochemical experiments: sham-operated, dMCAO, vehicle, and CA-074Me group. The animals from dMCAO group were sacrificed at 1, 2, 3, and 4 weeks, respectively, and the ones from the CA-074Me group were sacrificed at 2 weeks after operation. Single-labeled immunohistochemistry was conducted using the avidin-biotin-peroxidase complex (ABC) method. Briefly, the sections were rinsed with 0.01M PBS, treated with 3% H_2_O_2_ for 30 min, followed by 5% normal serum for 1 h at room temperature, and then incubated overnight at 4°C with the following primary antibodies: rabbit polyclonal anti-CathB (1:1000, Millipore, Cat# 06-480, **RRID**:AB_2292399), mouse anti-neuron-specific nuclear-binding protein (1:3000; Millipore, Cat# MAB377, **RRID**: AB_2298772), rabbit polyclonal anti-GFAP (1:3000, Millipore, Cat# AB5804, **RRID**: AB_11212369), and mouse anti-rat monocytes/macrophages (CD68) (1:1000; Millipore, Cat# MAB1435, **RRID**: AB_177576). After several PBS washes, the sections were incubated with biotinylated secondary immunoglobulin G antibody for 2 h at room temperature and were washed with PBS again, and then incubated with the ABC for 30 min at room temperature. The peroxidase reaction was visualized using the ABC method. Immunopositive cells in the SNr were quantified in three sections of each rat. The number of positive cells for NeuN, GFAP, and CD68 for each section was counted, respectively. Only cells with reaction products that presented within a clear and regular-shaped cytoplasmic border were quantified from three non-overlapping fields under 200× magnification and presented as the average cell number per field on each section. For densitometry analysis, the average intensity of CathB-positive staining in the SNr was determined using ImageJ software (NIH, Bethesda, MD, United States). Three non-repeated random fields under a light microscope (200×) in the SNr of each rat were assessed. These mean values were used for statistical analysis.

Triple-fluorescent immunohistochemistry was performed as previously described (Zhu et al., 2014). Sections were preincubated with 5% normal goat serum (containing 0.2% Triton X-100) for 1 h at room temperature, and then incubated overnight at 4°C with mixtures of rabbit and mouse primary antibodies: rabbit anti-CathB (1:100, Millipore, Cat# 06-480, **RRID**:AB_2292399), mouse anti-NeuN (1:1000; Millipore, Cat# MAB377, **RRID**: AB_2298772), mouse anti-GFAP (1:1000, Millipore, Cat# AB5804, **RRID**: AB_11212369), mouse anti-MAP-2 (1:1000, Millipore, Cat# MAB3418, **RRID**:AB_94856), and mouse anti CD68 (1:100; Millipore, Cat# MAB1435, **RRID**: AB_177576). After rinsing in 0.01M PBS, the sections were incubated for 1 h at room temperature with the following secondary antibodies: Cy3-conjugated goat anti-mouse IgG antibody (1:100; Millipore, Cat# AP124C, **RRID**: AB_11213281), and FITC-conjugated goat anti-rabbit antibody (1:50; Millipore, Cat# AP307F, **RRID**: AB_92652). After that, they were washed with PBS and mounted with mounting medium containing 4′,6-diamidino-2-phenylindole (DAPI). Slides were analyzed with a confocal laser microscope (Leica Microsystems, Wetzlar, Hessen, Germany).

### Western Blotting

The rats were sacrificed at 1, 2, 3, and 4 weeks after dMCAO, respectively. According to the “Rat brain in stereotaxic coordinates” (Paxinos and Watson, 2007), the brain tissue was cut into 2 mm-thick coronal slices using a brain matrix (Shuolinyuan Technology Co., Ltd., Beijing, China) and the ipsilateral SN (-4.8 to -6.04 mm, Bregma) was quickly dissected under a stereomicroscope. The proteins extracted from SN were separated with 15% SDS–PAGE gel, transferred to PVDF membranes, and incubated with antibodies against CathB (1:1000, Millipore, Cat# 06-480, **RRID**:AB_2292399), and glyceraldehyde-3-phosphate dehydrogenase (GAPDH 1:6000, Proteintech Group, Cat# 60004-I-Ig, **RRID**: AB_2107436) in Tris-buffered saline containing 0.2% Tween-20 (TBST) and 5% nonfat dry milk at 4°C overnight. Membranes were washed and incubated with the second antibody in TBST for 2 h. Densitometry analysis for the quantification of the bands was performed using image analysis software (ImageJ, NIH, Bethesda, MD, United States). Relative optical densities of protein bands were calibrated with GAPDH and normalized to those in sham-operated rats.

### Anterograde and Retrograde Tracing

In this study, anterograde and retrograde tracing were employed to label the neurons between the infarct cortex and the ipsilateral SN. Under anesthesia with 3–4% isoflurane, 10% biotinylated dextran amine (BDA) (10000 MW) (1 μL) were injected to the ipsilateral striatum of rats (0.4 mm posterior to the bregma, 3.0 mm lateral to the midline, and 5.0 mm below the dura) by stereotaxic instrument at 7 days of dMCAO. After the injection of BDA, the needle was maintained in place for 10 min before it was slowly extracted. Similarly, the attenuated (Bartha) strain of pseudorabies virus (PRV)-152, which was constructed to express EGFP (a gift from Enquist L. W., Princeton University), was used. PRV-152 viruses were grown in pig kidney (PK15) cells and stored at -80°C. The final titers deters determined in PK15 cells were 1 × 10^8-9^ pfu/ml for PRV152 (Xu et al., 2006).

Under anesthesia, 3 μl PRV-152 was injected to the ipsilateral SN of rats (5.4 mm posterior to the bregma, 2.1 mm lateral to the midline, and 8.3 mm below the dura) stereotactically at 7 days after dMCAO. The needle was left in place an additional 30 min being removed. A fresh stock of virus was thawed for each injection in this study.

### Pharmacologic Interventions

Implantation of an intracerebroventricular injection cannula into the right lateral ventricle was performed stereotaxically under anesthesia with 3–4% isoflurane. The cannula was placed through a burr hole opened on the right parietal skull at 1.5 mm lateral, 1.0 mm posterior, and 3.6 mm dorsal with respect to the bregma and affixed to the skull with stainless steel screws and cranioplastic cement. Rats were randomly divided into 3 groups (sham-operated, vehicle, and CA-074Me group). All rats were allowed to recover from surgery for 1 week before treatment. CA-074Me (16 nmol/d, Millipore, Bedford, MA, United States), or the vehicle [2% dimethyl sulfoxide (DMSO) in 0.01 M PBS] was injected into lateral cerebral ventricle once a day for 13 consecutive days; sham-operated rats received no interventional injection.

### The Evaluation of Neurological Function

Footprint analysis and open field test were performed at 1 and 2 weeks after dMCAO, respectively. The footprint test was conducted according to the procedure described by De Medinaceli et al. (1982). The forepaws and hindpaws of rats were stained with purple and blue dye. Rats were trained to walk through a 10-cm-wide, 60-cm-long corridor. Their footprints were recorded on the white absorbing paper. The average distance of steps was then measured and recorded. The open field test was performed following the procedure described by Dong et al. (2017) with a little modification. Rats were gently placed in the corner of a 100 cm × 100 cm × 35 cm uncowled plastic box and allowed to move freely in darkness for 10 min. The traveling distance and average speed of the freely moving rats were recorded using a video camera, and an experimenter blinded to the experimental conditions counted the total distance traveled by each rat. Smart 3.0 software (Panlab, Harvard Apparatus, Holliston, MA, United States) was used to analyze the videos.

### Data Analyses

Sample size in each group was determined prior to the execution of the experiments. All the experimental groups had at least five animals. Randomization was performed by using Random Number Table Method. Animals were divided randomly into different groups according to this standard procedure. After randomization, all analyses were performed blindly to group allocation. All continuous data are expressed as mean ± SD. and analyzed using SPSS 13.0 (SPSS Inc., Chicago, IL, United States). Statistical significance was determined by one-way analysis of variance (ANOVA), followed by LSD *post hoc* test or two-tailed Student’s *t*-test. *p* < 0.05 was considered statistically significant.

## Results

### Mortality

A total of 198 rats were used for the experiments. In the dMCAO groups, one rat died during the surgical procedure and five after surgery. A total of 7 rats died in the vehicle groups and 14 in the CA-074Me groups, which resulted from intracranial infection after intracerebroventricular injection. A total of 20 rats of neither neurologic deficit nor cortical infarction after dMCAO were excluded.

### The Gross Brain Morphology After dMCAO

Gross brain morphology confirmed the existence of focal cerebral infarctions in the dMCAO group, whereas no infarctions were observed in the sham-operated group (**Figures 1B,C**). Simultaneously, GFAP/DAB-staining assay in **Figure 1D** clearly showed that ischemic foci were confined within the cortex, while secondary degeneration occurred in the ipsilateral SN which was remote from the ischemic foci at 2 weeks after dMCAO, suggesting that the focal cortical infarction model was successfully developed. The relative infarction volumes were 15.89 ± 5.48% (*n* = 7), 16.38 ± 3.43% (*n* = 6), 17.41 ± 4.99% (*n* = 7), and 18.41 ± 4.62% (*n* = 6) in the 1, 2, 3, and 4 weeks after dMCAO, respectively. There was no significant difference in the infarct volume among the four groups (**Figure 1E**).

### Secondary Degeneration Was Observed in the Ipsilateral SN After dMCAO

**FIGURE 2:**
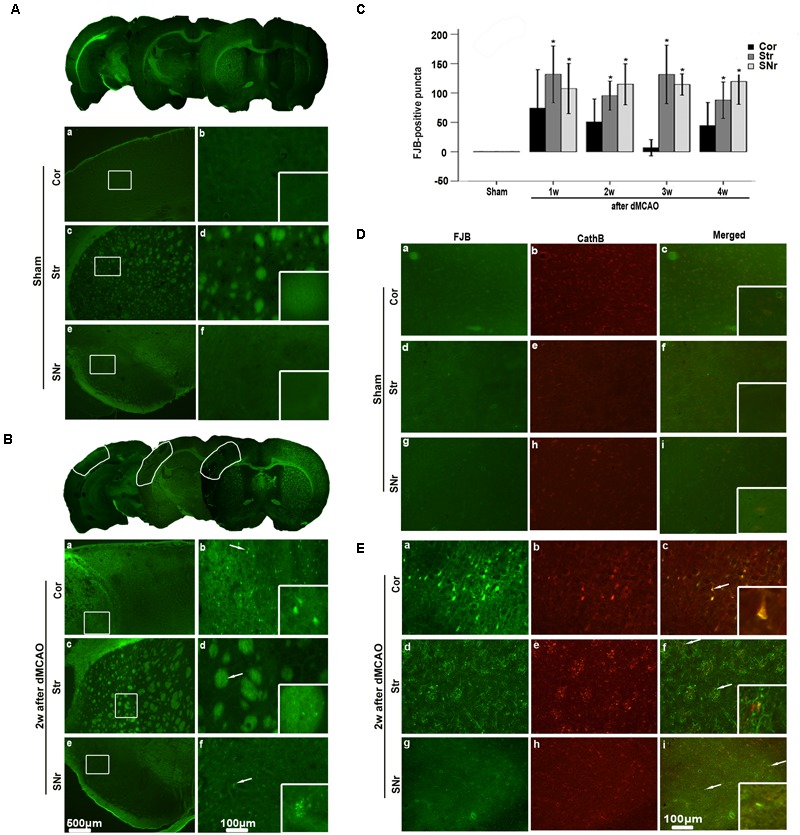
Secondary degeneration in the ipsilateral SNr after dMCAO in rats. **(A,B)** Representative microphotographs of FJB staining in the ipsilateral Cor, Str, SNr at sham operation **(A,a–f)** and at 2 weeks after dMCAO **(B,a–f)**. The panels at the bottom right corner are magnified from the outlined territory in the ipsilateral cortex, striatum, and SNr. Scale bar: **b,d,f**: 100 μm; **a,c,e**: 500 μm. **(C)** Quantitative analyses of FJB positive substance in the ipsilateral SNr at 1–4 weeks after dMCAO (*n* = 6, 6, 7, and 7, respectively) or sham operation (*n* = 6). Each bar represents the mean ± SD. ^∗^*p* < 0.05 versus sham-operated group. **(D,E)** Colocalization of CathB immunoreactive punctate and FJB positive substances at sham operation or after dMCAO. Representative images of fluorescent double-staining of CathB (red) and FJB positive substances (green) in the ipsilateral Cor, Str, SNr at sham-operated group and at 2 weeks after dMCAO. Cor, cotrex; Str, striatum; SNr, substantia nigra pars reticulata; Scale bar, 100 μm; w, week.

**FIGURE 3:**
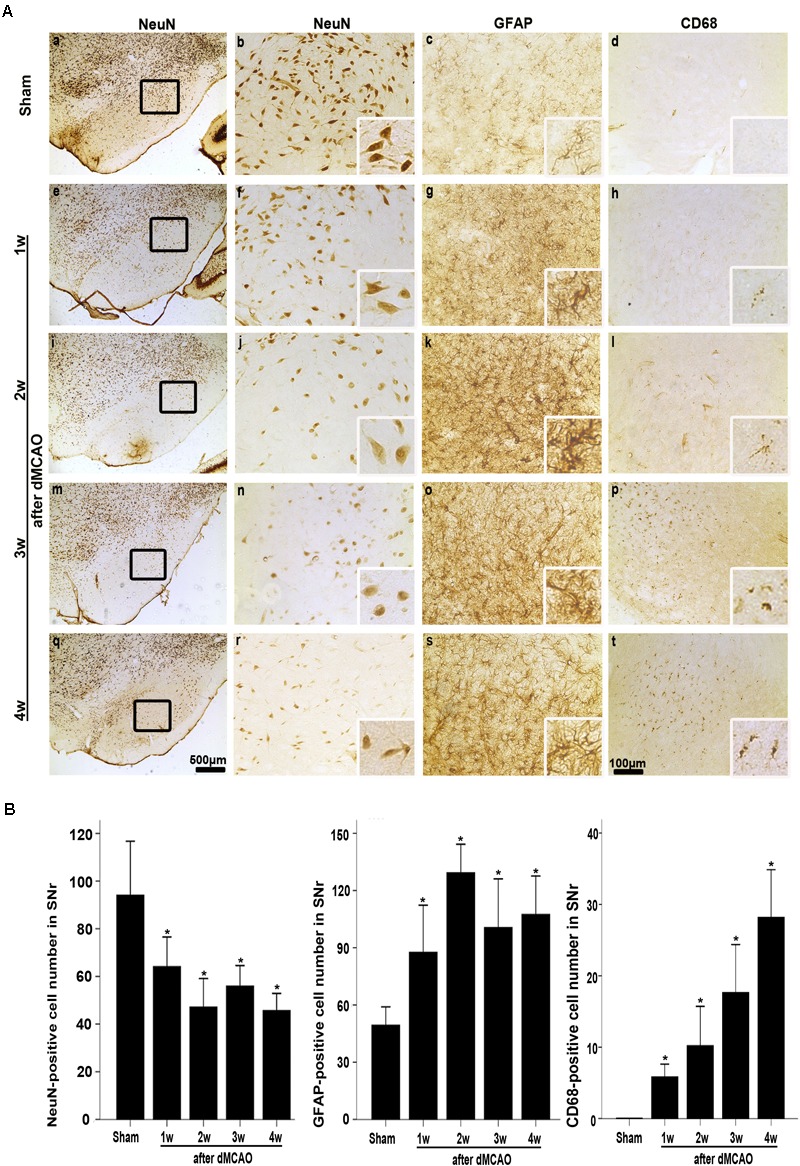
Secondary degeneration in the ipsilateral SNr after dMCAO in rats. **(A)** Representative microphotographs of immunostaining of NeuN, GFAP, and CD68 in the ipsilateral SNr at 1–4 weeks after dMCAO or sham operation. **a–d**, sham-operated group; **e–h**, 1 week after dMCAO; **i–l**, 2 weeks after dMCAO; **m–p**, 3 weeks after dMCAO; **q–t**, 4 weeks after dMCAO; Scale bar: **b–d,f–h,j–l,n–p,r–t**: 100 μm; **a,e,i,m,q**: 500 μm. **(B)** Quantitative analysis of NeuN-positive cells (*n* = 6 in sham-opreated group, *n* = 6, 6, 6, and 7 at 1–4 weeks after dMCAO, respectively), GFAP-positive cells (*n* = 6 in each group) and CD68-positive cells (*n* = 7 in sham-opreated group, *n* = 6, 7, 6, 6 at 1–4 weeks after dMCAO, respectively) in the ipsilateral SNr. Each bar represents the mean ± SD. ^∗^*p* < 0.05 versus sham-operated group. NeuN, neuron-specific nuclear-binding protein; GFAP, glial fibrillary acidic protein; CD68, cluster of differentiation 68; Sham, sham-operated; w, week.

The FJB positive cells or positive substances in the ipsilateral cortex, striatum, and substantia nigra pars reticulata (SNr) were observed all through in sham-operated animals (**Figures 2A,a–f**). Nonetheless, a substantial deposit of FJB positive puncta in the ipsilateral cortex, striatum, and SNr were observed at 2 weeks after dMCAO (**Figures 2B,a–f**). Quantitative analyses of FJB positive puncta showed that the axonal degeneration in the ipsilateral striatum and SNr all significantly accelerated from week 1 to week 4 after dMCAO compared to sham-operated group (**Figure 2C**). Moreover, CathB positive substances were observed in FJB positive cells or positive puncta in the ipsilateral cortex, striatum, and SNr at 2 weeks after dMCAO (**Figures 2D,E**). Progressive neuronal damage, characterized by the reduced number of NeuN-positive cells, was observed in the ipsilateral SNr. The numbers of NeuN-positive cell significantly decreased in the ipsilateral SNr from week 1 to week 4 after dMCAO when compared with the sham-operated group (**Figure 3B**). In contrast to the reduced number of neurons, the number of GFAP-positive cells from week 1 to week 4 and the number of CD68-positive cells from week 1 to week 4 after dMCAO at the same sites increased significantly when compared to the sham-operated group. These glial cells were characterized by their typical hypertrophic shape and thickened processes (**Figure 3A**). Thus, secondary degeneration in the ipsilateral SNr presented not only as profound neuronal loss, but also as extensive gliosis. However, no significant change in the number of NeuN-positive, GFAP-positive and CD68-positive cells was observed in the ipsilateral SNr of sham-operated rats (**Figure 3A**).

### CathB Was Upregulated in the Ipsilateral SN After dMCAO

**FIGURE 4:**
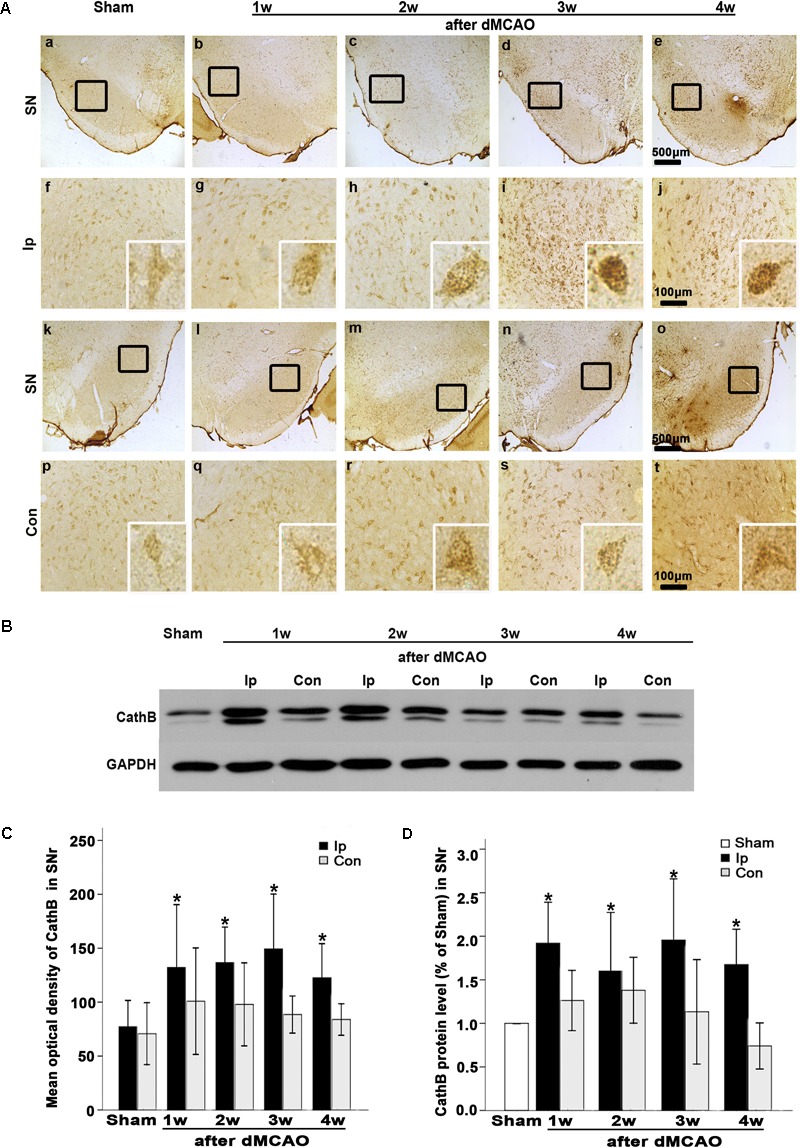
CathB was upregulated in the ipsilateral SNr after dMCAO. **(A)** Representative microphotographs of immunohistochemistry of CathB in the ipsilateral SNr at 1–4 weeks after dMCAO or sham operation: **a,f,k,p**, sham-operated; **b–e,g–j**, ipsilateral SNr after dMCAO; **l–o,q–t**, contralateral SNr after dMCAO. Scale bar: **a–e**: 500 μm; **f–o**: 100 μm. **(B)** Western blot analysis of CathB protein in the SN after dMCAO. **(C)** Quantitative analysis of CathB positive density in the SNr at 1–4 weeks after dMCAO (*n* = 7, 7, 6, and 7, respectively) and sham-opreated groups (*n* = 6). Data are expressed as percentage of value of sham-operated animals. Each bar represents the mean ± SD. ^∗^*p* < 0.05 versus the sham-operated animals. **(D)** Quantitative analysis of CathB protein in the SNr after normalization by GAPDH protein of the same sample. Data are expressed as percentage of value of sham-operated animals. Each bar represents the mean ± SD (*n* = 5 in each group). ^∗^*p* < 0.05 versus the sham-operated animals. GAPDH, glyceraldehyde 3-phosphate dehydrogenase; Ip, ipsilateral; Con, contralateral; Sham, sham-operated; w, week.

**FIGURE 5:**
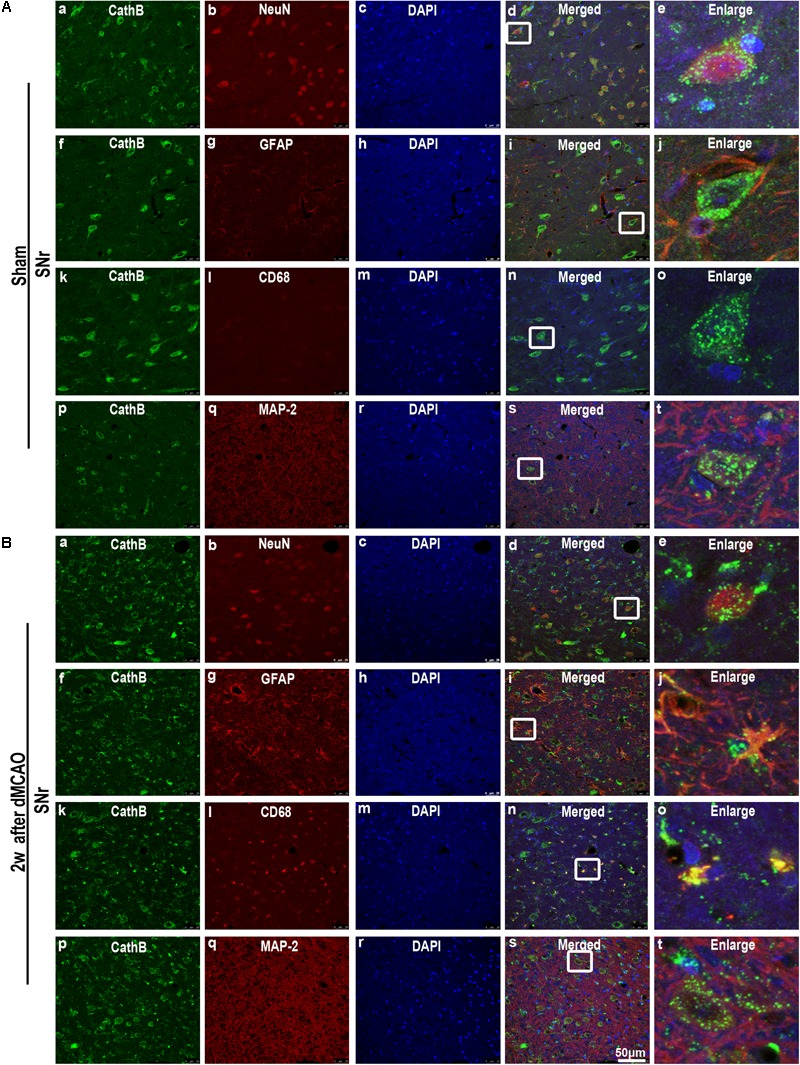
Cellular localization of CathB in the ipsilateral SNr after dMCAO. **(A)** Representative images of fluorescent triple-staining of CathB (green), DAPI (blue) and NeuN (red); CathB (green), DAPI (blue), and GFAP (red); CathB (green), DAPI (blue) and CD68 (red); CathB (green), DAPI (blue), and MAP-2 (red) in the ipsilateral SNr at sham-operated group. The overlapped images show that NeuN is surrounded by CathB **(a–e)**. No colocalization of CathB with GFAP **(f–j)**, CD68 **(k–o)**, and MAP-2 **(p–t)** was observed in the ipsilateral SNr at sham-operated group. **(B)** Representative images of fluorescent triple staining of CathB (green), DAPI (blue), and NeuN (red); CathB (green), DAPI (blue), and GFAP (red); CathB (green), DAPI (blue), and CD68 (red); CathB (green), DAPI (blue), and MAP-2 (red) in the ipsilateral SNr at 2 weeks after dMCAO. The overlapped images show that NeuN is surrounded by CathB **(a–e)**; and CathB is colocalized with GFAP **(f–j)**, CD68 **(k–o)**, and MAP-2 **(p–t)** in the ipsilateral SNr at 2 weeks after dMCAO. Scale bar, 50 μm. NeuN, neuron-specific nuclear-binding protein; GFAP, glial fibrillary acidic protein; MAP-2, microtubule-associated proteins-2; CD68, cluster of differentiation 68; Sham, sham-operated; w, week.

In sham-operated group, immunoreactive substances of CathB were found mainly in the cells with round nuclei and spindle cell body and elongated axons, which showed a typical neuron-like morphology (**Figures 4A,a,f,k**). Triple labeling with immunofluorescence assay further confirmed that these cells were NeuN-positive (**Figures 5A,a–e**) and MAP-2 positive (**Figures 5A,p–t**), indicating a predominantly neuronal localization of CathB in sham-operated brains. However, CathB granules in the ipsilateral SNr became progressively larger and irregular and formed aggregates or diffuse cytoplasmic staining from 1 to 4 weeks after dMCAO, suggesting that CathB has escaped from the lysosome containment [**Figures 4A(g–j), 5B(a–e)**]. Quantitative analysis of CathB positive density showed that the expression of CathB in the ipsilateral SNr was increased after dMCAO (**Figure 4C**). Consistently, similar results were observed by Western blotting (**Figures 4B,D**). Moreover, triple labeling revealed that CathB positive substances were either GFAP or CD68 positive (**Figures 5B,f–o**), indicating that CathB in the ipsilateral SNr was also localized in astrocytes and microglia at 2 weeks after dMCAO.

### Inhibition of CathB Enhanced the Cortico-Striatum-Nigral Connections After dMCAO

**FIGURE 6:**
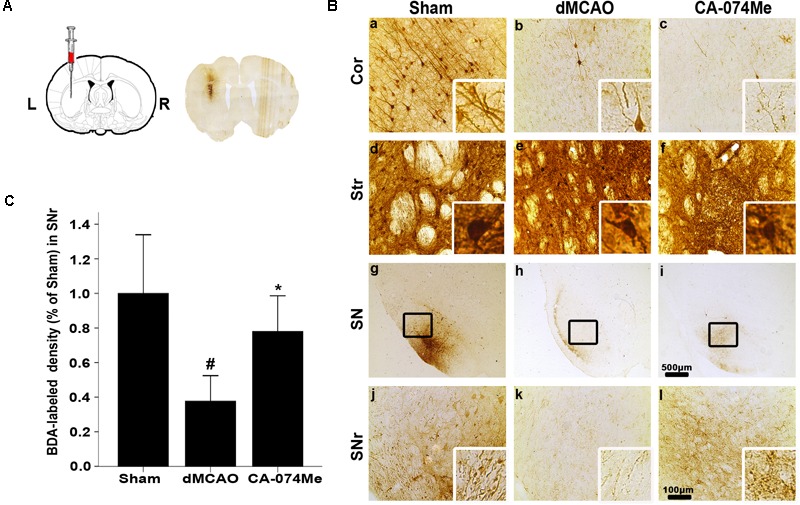
Cortico-striatum-nigral tract tracing with BDA. **(A)** The representative image of injection site. **(B)** Representative microphotographs of histochemical photographs displayed BDA-labeled in the ipsilateral cortex, striatum, and SNr at sham-operated group (*n* = 5) **(a,d,g,j)**, dMCAO group (*n* = 5) **(b,e,h,k)**, and CA-074Me group (*n* = 6) **(c,f,i,l)**. **(C)** Quantitative analysis of BDA-labeled density in the SNr after dMCAO. Data are expressed as percentage of value of sham-operated animals. Each bar represents the mean ± SD. ^∗^*p* < 0.05 versus the dMCAO animals. ^#^*p* < 0.05 versus the sham-operated group. Scale bar: **a–f**, **j–l**: 100 μm; **g–i**: 500 μm. Cor, cotrex; Str, striatum; SN, substantia nigra; SNr, substantia nigra pars reticulata; Sham; sham-operated.

**FIGURE 7:**
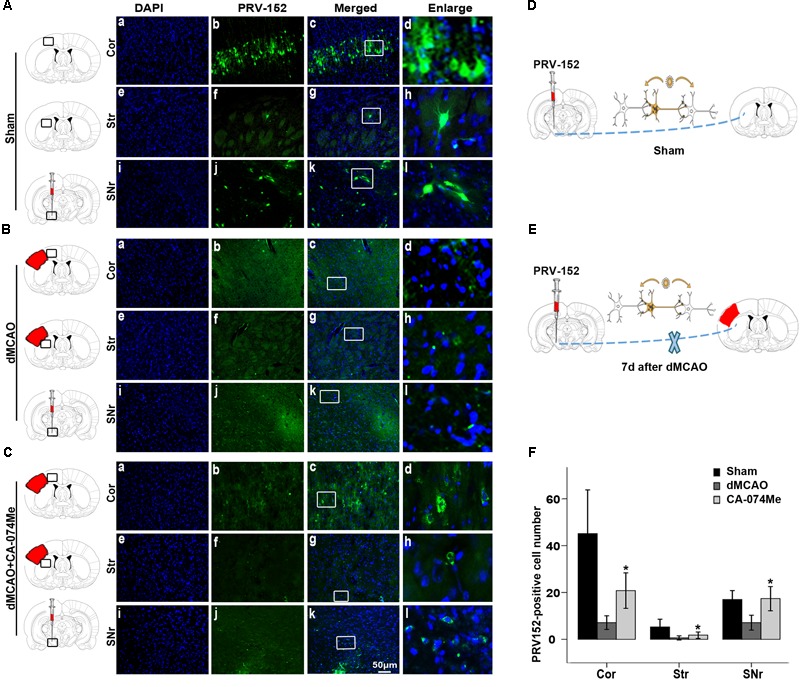
Cortico-striatum-nigral tract tracing with PRV-152. **(A)** Photographs showed that PRV-152 (green) was injected into the ipsilateral SNr **(i–l)** and its signals were observed in the pyramidal neurons in the ipsilateral frontal and parietal cortex **(a–d)** and striatum **(e–h)** at 4 days after injection in sham-operated group (*n* = 6). **(B)** Photographs showed that PRV-152 (green) was injected into the ipsilateral SNr **(i–l)** and its signals were observed in the pyramidal neurons in the ipsilateral frontal and parietal cortex **(a–d)** and striatum **(e–h)** at 4 days after injection in dMCAO group (*n* = 7). **(C)** Photographs showed that PRV-152 (green) was injected into the ipsilateral SNr **(i–l)** and its signals were observed in the pyramidal neurons in the ipsilateral frontal and parietal cortex **(a–d)** and striatum **(e–h)** at 4 days after injection in CA-074Me group (*n* = 7). Scale bar, 50 μm. **(D–E)** Schematic illustration of the fluorescent signal of PRV-152 in sham-operated group and dMCAO group after PRV-152 was injected into the ipsilateral SNr. **(F)** Quantitative analysis of PRV152-positive cell number in the cortex, striatum, and SNr after dMCAO. Each bar represents the mean ± SD. ^∗^*p* < 0.05 versus the dMCAO animals. Cor, cotrex; Str, striatum; SNr, substantia nigra pars reticulata; Sham; sham-operated; d, day.

To study whether the inhibition of CathB enhanced cortico-striatum-nigral connections after dMCAO, BDA, or PRV-152 for anterograde, or retrograde tracing was injected stereotaxically to striatum or SN. The results showed that the administration of 16 nmol CA-074Me by intracerebroventricular injection starting at 24 h after dMCAO increased the density of BDA-labeled fibers in the ipsilateral SNr (**Figure 6**). Moreover, CA-074Me treatment increased the numbers of PRV-152 positive-cell in the striatum and cortex in the CA-074Me group after focal cortical infarction when compared to the dMCAO group (**Figure 7**). The results suggested that the inhibition of CathB potentially enhanced cortico-striatum-nigral connections after dMCAO.

### Inhibition of CathB Alleviated Secondary Degeneration in the Ipsilateral SNr After dMCAO

**FIGURE 8:**
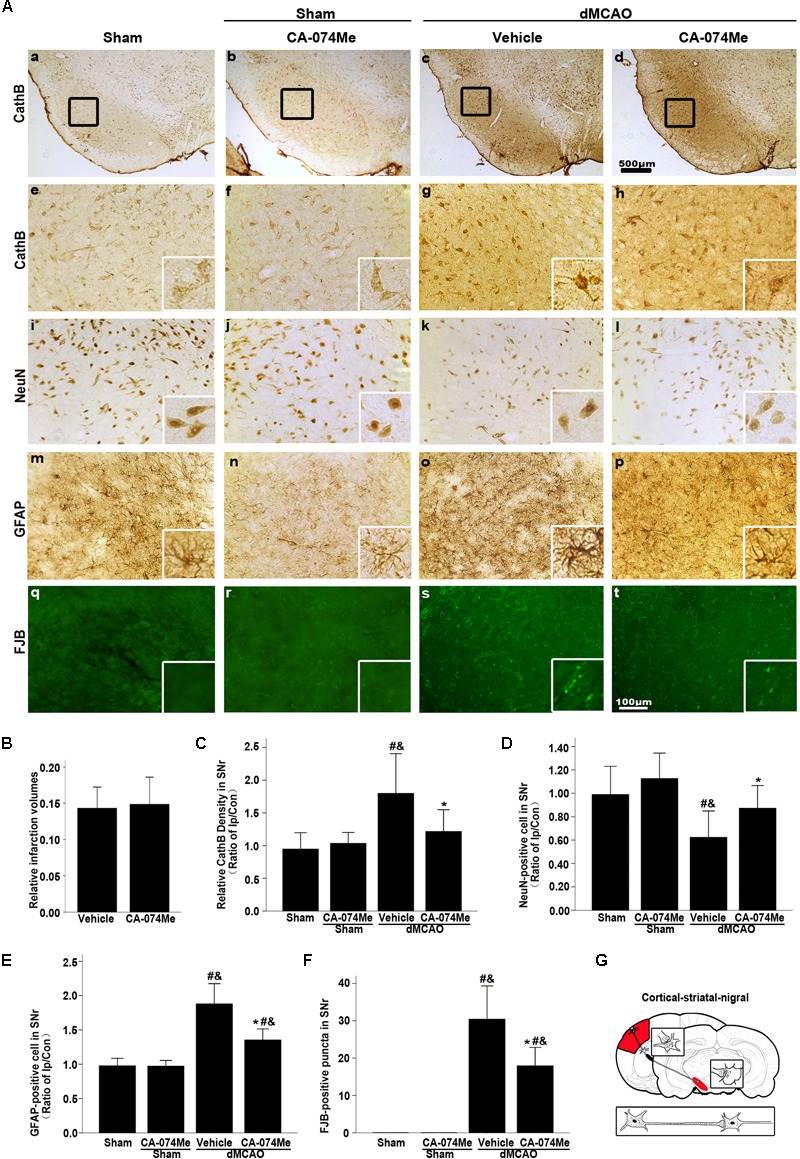
Delayed CA-074Me treatment alleviated secondary degeneration of ipsilateral SNr after dMCAO. **(A)** Representative microphotographs of immunostaining of CathB, NeuN, GFAP, and FJB staining in the ipsilateral SNr. Scale bar: **e–t**: 100 μm; **a–d**: 500 μm; **a,e,i,m,q**, the ipsilateral SNr in sham-operated group; **b,f,j,n,r**, the ipsilateral SNr in sham-operated group with CA-074Me; **c,g,k,o,s**, the ipsilateral SNr in the vehicle group after dMCAO; **d,h,l,p,t**, the ipsilateral SNr in the CA-074Me group after dMCAO. **(B)** Relative infarction volumes in the vehicle (*n* = 6) and CA-074Me (*n* = 6) groups after dMCAO. **(C)** Quantitative analysis of CathB-positive density in the SNr at sham-operated group (*n* = 5), sham-operated with CA-074Me group (*n* = 5), vehicle (*n* = 6), and CA-074Me (*n* = 8) groups after dMCAO. **(D)** Quantitative analyses of NeuN-positive cells in the ipsilateral SNr at sham-operated group (*n* = 5), sham-operated with CA-074Me group (*n* = 5), vehicle (*n* = 6), and CA-074Me (*n* = 8) groups after dMCAO. **(E)** Quantitative analyses of GFAP-positive cells in the ipsilateral SNr at sham-operated group (*n* = 5), sham-operated with CA-074Me group (*n* = 5), vehicle (*n* = 6), and CA-074Me (*n* = 7) groups after dMCAO. **(F)** Quantitative analyses of FJB-positive substance in the ipsilateral SNr at sham-operated group (*n* = 5), sham-operated with CA-074Me group (*n* = 5), vehicle (*n* = 7), and CA-074Me (*n* = 6) groups after dMCAO. Data was expressed as the ratio of ipsilateral/contralateral. Each bar represents the mean ± SD. ^∗^*p* < 0.05 versus the vehicle group. #*p* < 0.05 versus the sham-operated group; &*p* < 0.05 versus the sham-operated with CA-074Me group. **(G)** Schematic illustration of cortico-striatum-nigral pathway in rats after dMCAO. NeuN, neuron-specific nuclear-binding protein; GFAP, glial fibrillary acidic protein; FJB, Fluoro-Jade B; Ip, ipsilateral; Con, contralateral; Cor, cotrex; Str, striatum; SNr, substantia nigra pars reticulata; Sham; sham-operated.

At 2 weeks after dMCAO, relative infarct volume computed from GFAP/DAB-staining sections in vehicle-treated rats was 14.32% ± 2.78% (*n* = 6), and 14.86% ± 4.05% (*n* = 7) in CA-074Me rats. There was no significant difference in the infarct volume between the two groups. Intracerebroventricular administration of 16 nmol CA-074Me starting at 24 h after dMCAO significantly decreased the expression of CathB in the ipsilateral SNr (**Figures 8A,a–h**). Accordingly, in the ipsilateral SN, the number of NeuN-positive cells was significantly higher (**Figure 8D**), while the number of GFAP positive cells and FJB positive substances were significantly lower (**Figures 8E,F**) in the CA-074Me group at 2 weeks after dMCAO when compared with the vehicle group. As shown in **Figure 8**, CA-074Me had no any neurotoxic effect in SNr in sham-operated rats. Taken together, these results suggested that the inhibition of CathB alleviated post-dMCAO secondary degeneration in the ipsilateral SNr.

### The Evaluation of Neurological Function After Focal Cortical Infarction

**FIGURE 9:**
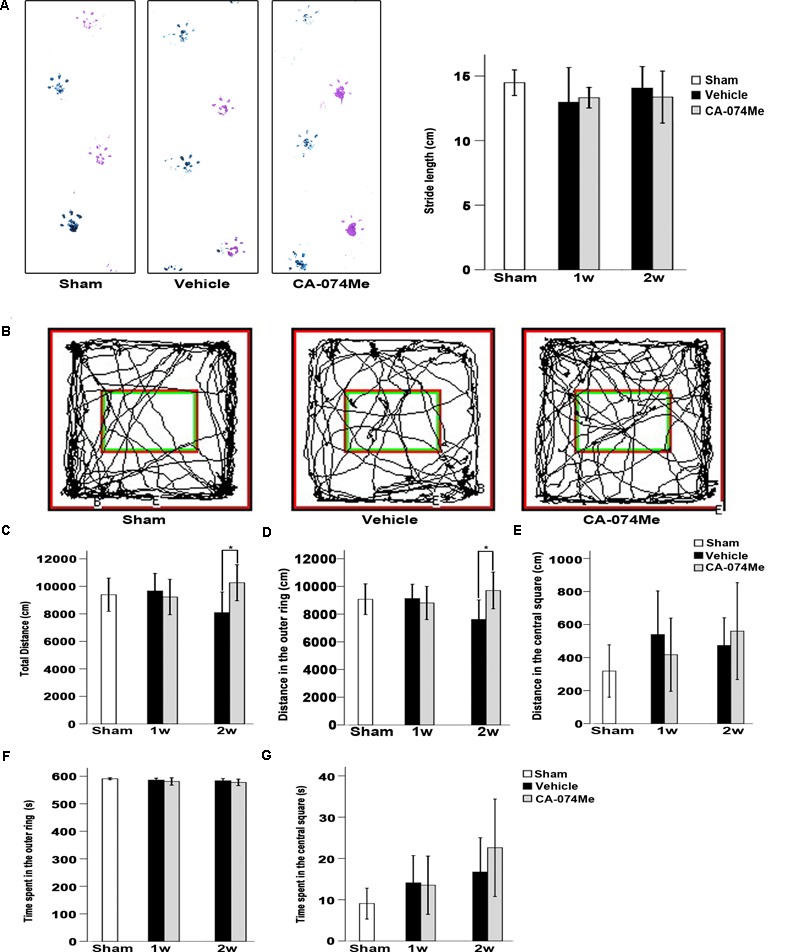
Neurological function evaluation. **(A)** Typical footprints of animal walking at 2 weeks after dMCAO. Blue: Left forepaw and hindpaw footprints; Purple: Right forepaw and hindpaw footprints; the average footstep length was quantified. Each bar represents the mean ± SD. No statistically differences were observed between vehicle and CA-074Me groups after dMCAO compared to sham-operated group (*n* = 10) at 1 week (*n* = 7 and *n* = 9 for vehicle and CA-074Me groups after dMCAO, respectively) and 2 weeks (*n* = 9 and *n* = 8 for vehicle and CA-074Me rats after dMCAO, respectively). **(B)** Representative tracks for open field test at sham-operated group, vehicle, and CA-074Me group after dMCAO; the total distance traveled **(C)** and its distance traveled in the outer ring **(D)**, the central square **(E)** in sham-operated (*n* = 8), vehicle (*n* = 7), and CA-074Me (*n* = 8) treated rats after dMCAO during 10 min in the open field. The duration of walk in the outer ring **(F)** and the central square **(G)** in sham-operated (*n* = 8), vehicle (*n* = 7), and CA-074Me (*n* = 8) treated rats after dMCAO during 10 min in the open field. Each bar represents the mean ± SD. ^∗^*p* < 0.05 versus the vehicle group. Sham; sham-operated; w, week.

The walking pattern of rats was evaluated through footprint analysis of gait (**Figure 9A**). The average footstep lengths were 14.48 ± 1.38 (*n* = 10) in sham-operated group, 12.97 ± 2.91 in vehicle (*n* = 7) and 13.32 ± 1.03 in CA-074Me (*n* = 9) groups at 1 week after dMCAO, 14.07 ± 2.18 in vehicle (*n* = 9) and 13.37 ± 2.40% in CA-074Me (*n* = 8) groups at 2 weeks after dMCAO, respectively. There were no significant differences in the average footstep length among these groups. The spontaneous motor activity of rats was also assessed in an open field enclosure (**Figure 9B**). Our results showed that CA-074Me significantly increase the total distance traveled and the distance traveled in the outer ring at 2 weeks after dMCAO when compared to the vehicle group (**Figures 9C,D**), but not in the central square (**Figure 9E**). There was no significant difference between CA-074Me and vehicle groups in the time spent in the outer ring and in the central square in open field test (**Figures 9F,G**).

## Discussion

The present study confirms that secondary degeneration occurred in the ipsilateral SN after dMCAO in adult SD rats. Moreover, to our best knowledge, this is the first work to demonstrate that CathB involved in the aforementioned process, and that the inhibition of CathB with Ca-074Me enhances the cortico-striatum-nigral connections and exerts neuroprotection against secondary degeneration in the ipsilateral SN after focal cortical infarction.

During the process of sudden cortical cerebral blood supply disturbance, secondary degeneration may occur at distant sites separate from the dominant vessels. It has been documented that secondary degeneration in the ipsilateral SNr after focal cortical stroke, in rodents, nonhuman primates, and humans (Ohe et al., 2013; Rodriguez-Grande et al., 2013; Chen et al., 2015; Prinz et al., 2015). In this study, we electrocoagulated the MCA distal to striate branch, similar to what Xing et al. (2012) did in rodent model. The blood supply of SN in the rat is from penetrating branches of posterior cerebral artery and superior cerebellar arteries. Thus, occlusion of the distal MCA to the striate branches does not affect the blood supply of SN. We found the significant increases of FJB positive puncta in the ipsilateral striatum and SNr, neuronal loss, and gliosis in the ipsilateral SNr after focal cortical infarction sequentially and they progressed over time. Our present findings confirmed substantial progressive secondary degeneration in the ipsilateral SNr after dMCAO, which is remote from the primary ischemic infarction in the cortex.

Up till now, the underlying molecular mechanisms of secondary degeneration in the ipsilateral SN after dMCAO have not been completely elucidated. Retrograde degeneration, anterograde degeneration, and transneuronal degeneration might be the major mechanism accounting for the development of secondary degeneration in the ipsilateral SN after focal cortical stroke. Cysteine proteases, known as thiol proteases, are enzymes that degrade proteins. CathB is one of the major neuronal lysosomal proteases that plays key roles in intracellular protein catabolism (Olson and Joyce, 2015), autophagosome formation (Shen and Mizushima, 2014; Fekadu and Rami, 2016), and axon outgrowth (Pišlar and Kos, 2014). However, under pathological stimulation, Cath are thought to cause cellular autolysis and damage to neighboring cells during necrosis (Liu et al., 2014; Xu et al., 2016), and to act as mediators of programmed cell death (Brojatsch et al., 2015; Ge et al., 2016). It has been reported that CathB was involved in apoptotic regulation via cleaving Bid to tBid, leading to permeabilization of the outer mitochondrial membrane, release of cytochrome-c into the cytosol, and activation of caspase3 (Xu et al., 2014; Zuo X. et al., 2016). Increasing evidences show that CathB takes part in the process of axon degeneration (Manninen et al., 2014). In this study, we first investigated the roles of CathB in the formation of secondary degeneration in the ipsilateral SN after focal cortical infarction. The expression of CathB in the ipsilateral SNr was significantly increased after dMCAO, which would result in lysosomal CathB leakage to cytosol. In addition, our results revealed that, more CathB positive substances colocalized with FJB positive cells or positive puncta in the ipsilateral cortex, striatum, and SNr at 2 weeks after dMCAO compared to sham-operated group, indicating that activated CathB was indeed associated with the secondary degeneration and it played an important role in injuries spread through cortico-striatum-nigral projections after focal cortical stroke. Interestingly, CathB in the ipsilateral SNr was also localized in astrocytes and microglia at 3 weeks after dMCAO. Previously, glial cells were reported not to synthesize CathB until cerebral ischemia begins (Zuo X. et al., 2016), whereas neurons are constitutively rich in CathB. In the meantime, glial CathB is more likely to be secreted by its host into the extracellular space to induce neuronal apoptosis (Kingham and Pocock, 2001), rather than functioning intracellularly as does neuronal CathB (Benchoua et al., 2004; Sakamoto et al., 2008; Hwang et al., 2009; Terada et al., 2010; Wu et al., 2017). In addition, it was reported that the activation of CathB could conduce to the processing and releasing of IL-1β in microglia, whereas blockade of CathB activity with the inhibitor CA-074Me markedly attenuated IL-1β release in a dose-dependent manner from wild-type, NOD-like receptor family, pyrin domain containing 3 (NLRP3) knockout, and apoptosis-associated speck-like protein (ASC) knockout microglia (Hanamsagar et al., 2011). These studies demonstrated that CathB in glial cells exerts crucial influences in neuroinflammation or neurodegenerative disorder, etc. In this study whether glial CathB contributes to the secondary degeneration in the ipsilateral SNr after focal cerebral infarction, further experimental study is needed to conduct.

Based on previous study (Hook et al., 2015) and our results, selective inhibitor of CathB would be a powerful tool for clarifying the functions of CathB. Membrane-permeable CathB inhibitor CA-074Me has been used widely to inactivate CathB *in vivo* and *in vitro* (Steverding, 2011; Cho et al., 2013; Gao et al., 2014; Zhang et al., 2015; Zuo X. et al., 2016). It has been demonstrated that the inhibition of CathB by CA-074Me has neuroprotective effects against cerebral ischemic injury (Xu et al., 2014; Hook et al., 2015; Xu et al., 2016). SN is an important neuronal structure, located in the ventral midbrain and adjacent to the substantia nigra pars compacta (SNc), and exerts a regulatory function within the basal ganglia circuitry through the nigro-striatal pathway. SNr is the afferent hub of SN, especially for those inputs from the striatum. It is mainly composed of γ-aminobutyric acid (GABA) neurons, which provides a direct inhibition to glutamatergic thalamo-cortical neurons. Also, the activity of SNr cells is regulated by inhibitory and excitatory inputs arising in a variety of brain areas (Guatteo et al., 2009). After cortical cerebral ischemia the glutamatergic and GABAergic imbalance occurs and may bring about excitotoxicity to the SN neurons through retrograde and anterograde degeneration in the cortico- striatum-nigral pathway. However, the mechanisms underlying secondary degeneration in the SN after focal cortical infarction are complex and poorly understood. So far, the existence of a direct cortico-nigral pathway has been demonstrated in the rodent (Zakiewicz et al., 2014) and human brain (Cacciola et al., 2016). These results may be relevant for the comprehension of the pathophysiology of secondary degeneration in the SN after focal cortical infarction. Preservation of neural networks is the fundamental anatomic basis for the recovery of function in mammalian brain after stroke. Failure of it will lead to failure of recovery. Therefore, we investigated whether the inhibition of CathB could enhance cortico-nigral connections after dMCAO. We traced striatum-nigral and cortico-nigral projections with BDA or PRV-152. Our results suggested that the inhibition of CathB could enhance cortico-striatum-nigral connections after dMCAO. Theoretically, enhancing cortico-striatum-nigral connections by inhibition of CathB should be beneficial for the post-stroke functional recovery. Further studies to uncover the mechanism of neural networks abnormalities after stroke are necessary. Fortunately, advances in neuroimaging techniques will soon allow us to understand this mechanism at multiple levels (Rondina et al., 2016; Ward, 2017).

Previously, we have reported that the activation of CathB in the VPN was involved in apoptotic regulation through cleaved caspase-3 and CA-074Me exerted neuroprotection against secondary degeneration after dMCAO (Zuo X. et al., 2016). It is possible that cortical cerebral infarction causes lysosomal ruptures and the release of CathB into the cytosol, then triggers apoptosis or apoptosis-like death via cleaving Bid, leading to permeabilization of the outer mitochondrial membrane, release of cytochrome-c into cytosol, and activation of caspase cascade (Xu et al., 2014; Kim et al., 2015; De Castro et al., 2016). In the present study, CA-074Me might reduce translocation of CathB from the lysosomes into the cytoplasm, inactivate caspase-3 through inhibiting CathB activity in the ipsilateral SNr, enhance cortico-striatum-nigral connections, and alleviate secondary degeneration in the ipsilateral SN, eventually improve neurological function to some extent after dMCAO.

## Conclusion

The present study demonstrated that secondary degeneration in the ipsilateral SN after dMCAO in adult rats was mediated and progressed by CathB. Targeting CathB with inhibitor CA-074Me may enhance the cortico-striatum-nigral connections and offer a potential therapeutic strategy of neuroprotection against secondary degeneration of remote areas for focal cerebral ischemic stroke with an extended window.

## Author Contributions

EX and QH conceived and designed the experiments. XZ, JJ, LZ, ZS, YT, and WS performed the experiments. XZ, JJ, LZ, XC, and WS collected the data and processed them. XZ and EX wrote the paper. All authors read and approved the manuscript.

## Conflict of Interest Statement

The authors declare that the research was conducted in the absence of any commercial or financial relationships that could be construed as a potential conflict of interest.
